# Body-centered encoding of passive tactile pattern memories

**DOI:** 10.1038/s41598-026-52275-3

**Published:** 2026-05-28

**Authors:** Shreyas Indurkar, Betül Kayacik, Peng Liu, Esther Kuehn

**Affiliations:** 1 Hertie Institute for Clinical Brain Research (HIH), University of Tübingen, Tübingen, Germany; 2 German Centre for Neurodegenerative Diseases (DZNE), Tübingen, Germany

**Keywords:** Episodic memory, Multisensory integration, Explicit memory, Vibrotactile stimulation, Contextual learning, Tactile discrimination, Passive touch, Neuroscience, Psychology, Psychology

## Abstract

The human brain stores and retrieves tactile experiences, allowing object recognition by touch, the definition of haptic preferences, and the retrieval of past bodily experiences. However, little is known about the spatial code of tactile body memories, particularly whether encoding takes place in a body-centered (tactile) reference frame, not influenced by hand posture or visual cues, or whether it takes place in an external reference frame, where tactile information is integrated with proprioceptive and visual information. Here, we combined a passive tactile pattern memory task with the crossed-hands paradigm to investigate if tactile pattern retrieval accuracy is influenced by in-/congruent hand position during learning and retrieval (experiment 1) and/or the spatial context surrounding the hand (experiment 2). We hypothesized that significant effects of hand position and/or visual context on retrieval accuracy evidence external encoding, whereas the absence of such effects are more consistent with body-centered encoding. Our data is in accordance with the latter hypothesis, and do not support external encoding. The results can be considered plausible in light of clinical evidence where bodily sensations related to past memories are often confined to specific body locations rather than remapped to external space.

## Introduction

The human brain is equipped with the ability to perceive, store and retrieve tactile experiences that we feel on the body. This allows us to identify objects by touch^1^ and to remember emotional tactile experiences^2^. Tactile memories define our haptic preferences and emotional associations with bodily touch, and can manifest as mental health problems, such as somatic symptoms or flashbacks as part of trauma disorders^3,4^. Yet, our scientific knowledge on the neuronal mechanisms that underlie the memory of tactile experiences in humans is so far limited. Specifically, the spatial code of tactile memories is so far not fully clarified.

The spatial code of tactile memories defines whether encoding and retrieval are based only on body-centered (tactile) information, or whether tactile information is integrated with proprioceptive (position) and/or visual information when this information is available^5,6^. Tactile information is first relayed to the thalamus and area 3b of the primary somatosensory cortex (SI) following the principles of homuncular organization in a somatotopic format^7–10^. Body-centered encoding has, in this context, been defined as the representation of the stimulated body location within the homuncular organization of area 3b, which is at initial processing stages to a large extent independent of body posture^5,11,12^. When this information is integrated into posterior networks, specifically area 2, area 5, and the the posterior parietal cortex (PPC), it is increasingly remapped into an external reference frame, which allows to localize the tactile stimulation in extrapersonal space^13^. During this remapping, somatosensory information stemming from tactile receptors is integrated with body posture (proprioception) and associated visual information, such as the relation between the body and the environment^11^. The importance of the parietal cortex for tactile memories has been outlined several times^14,15^. One hypothesis on the spatial code of tactile memories is that tactile information is encoded, memorized and retrieved in an external reference frame, in which body-centered information is integrated with external information.

However, SI is also connected to the secondary somatosensory cortex (SII) and insular cortex, which are assumed to contribute to the storage and retrieval of tactile memories. Specifically the connection between the insular cortex and the perirhinal cortex in the medial temporal lobe (MTL) is considered important, as associated limbic structures facilitate the formation of sensory memory traces^16^. The storage of tactile information may therefore rely on the interaction between SI, SII, the insula, and the structures of the MTL for long-term memory^17^. It has been previously shown that not only SI and SII, but also the insula cortex has somatotopic representational schemes less influenced by body position^18–21^. SI, SII and the insular cortex are also involved in the processing of tactile memories, as shown in fMRI studies^22–24^. Based on these findings, one could also hypothesize body-centered encoding and retrieval of tactile information via this pathway.

One study addressed the question of whether tactile memories are encoded in a body-centered or an external reference frame^25^. Blindfolded participants were asked to memorize a tactile map with palpable raised lines by scanning it using their hands, and to recall the landmarks when prompted. Four conditions were tested: (1) tactile memory in a body-centered reference frame, with the map aligned to the participant’s midline, (2) disruption of the body-centered reference frame by rotating the map, (3) memory in an external reference frame, with a palpable raised frame added around the map as a reference, and (4) memory when both the body-centered alignment and external frame were added. The authors reported that participants’ performance decreased significantly when the body-centered reference frame was disrupted. The performance improved significantly, however, with the addition of the external frame, concluding that both body-centered reference and external reference frames can influence the spatial coding of tactile cues. This is inline with an fMRI study on working memory of tactile stimuli reporting that SI retains the tactile information whereas PPC retains the spatial information^26^. However, in this experiment, participants actively explored the stimuli using finger and hand movements, thereby integrating body movement and touch already during learning. The spatial code of passive tactile memories is therefore not targeted in these studies.

Unlike active touch employed in the above-mentioned experiment, where participants actively navigated their finger on the map, we used a passive touch paradigm, where stimulation was given to the participants without any finger or body movement. To investigate whether tactile pattern memories, acquired via passive touch without body movement, are encoded in a body-centered or external reference frame in a situation where proprioceptive and visual information are task-irrelevant, we combined tactile pattern learning with the established crossed-hands paradigm. PPC activation has been evidenced in an fMRI study using the crossed-hands paradigm^27^, and it allows to test interference effects between tactile stimuli learned in a crossed or uncrossed hand position with congruent or incongruent retrieval. Indeed, in the crossed-hands paradigm, hand position interferes with tactile encoding even when this information is a distraction (i.e., slower reaction times in uncrossed condition). Transferring the crossed-hands paradigm to a tactile pattern memory study allowed us to target two specific questions: (Q1) Is retrieval accuracy of learned tactile patterns at the fingertip influenced by hand position (i.e. proprioception)? (Q2) Is retrieval accuracy of learned tactile patterns at the fingertip influenced by visual context? Q1 specifically targets the influence of in/congruent proprioceptive information on tactile pattern retrieval, whereas Q2 addresses the additional input of visual context information during retrieval, either congruent or incongruent to the learned stimulus. We hypothesised that, if tactile memories are encoded in an external reference frame, where tactile, proprioceptive and visual information is integrated, the accuracy of pattern recall would be significantly higher in congruent compared to incongruent conditions (i.e., when learning and retrieval takes place in the same hand position, and within the same visual context). Conversely, if there is no significant difference between conditions, this would not support external encoding but would be consistent with body-centered (tactile) encoding, because in this case, the accuracy of pattern recall depends on representations that are not significantly influenced by vision and proprioception.

The current study helps to clarify the spatial code in which passively perceived tactile information is stored and retrieved, providing valuable insight into the cognitive mechanisms underlying human tactile memories and associated clinical conditions.

## Results

**Fig. 1:**
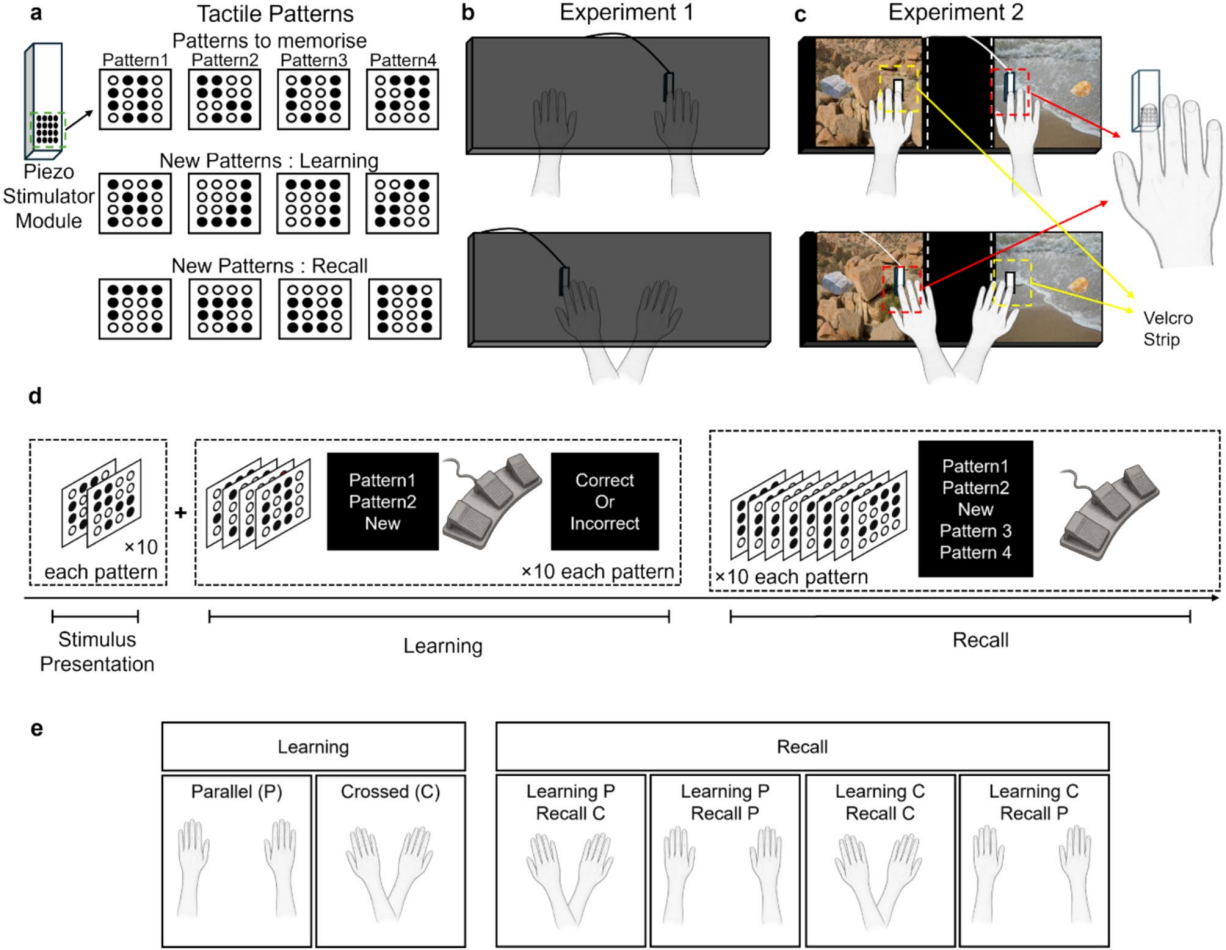
Overview of experimental setup. In both experiments, participants were asked to learn and memorize tactile patterns presented to their right index fingertip with their hands either in parallel (P) or crossed (C) positions. (**a**) Piezo stimulator module (manufactured by Quaerosys) and tactile patterns used for learning. 4 patterns were memorized by participants during stimulus presentation, 4 new patterns were presented together with the learned pattern during discrimination learning. 4 more new patterns were presented together with the 4 learned patterns during recall. (**b**) In experiment 1, the participants’ hands were occluded from view with an opaque black cardboard box. (**c**) In experiment 2, participants’ hands were visible and placed on a monitor. The monitor was laid screen-up in front of the participant, where images were shown during stimulus presentation, learning and recall phases, providing rich visual context. A stone and seashell were placed on the screen additionally to provide 3D context. (**d**) The experiment started with the presentation of 4 tactile patterns; each pattern was presented to the participant’s index finger 10 times, with hands in positions P or C. The hand was placed palm down and the tactile pattern was not visible. In each run, 2 patterns were presented in succession. In the learning phase, the memorized patterns were recalled with feedback (i.e., participants were shown if they had identified the patterns correctly), with hands in position P or C (same position as during stimulus presentation). Stimulus presentation and learning were repeated for the other two patterns. During the recall phase, each pattern was recalled without feedback (i.e., participants were not shown if their response was correct). In this phase, each pattern was recalled with hands in both positions P and C, leading to either congruent or incongruent recall with respect to hand position only (experiment 1: proprioception), or learning position and visual context (experiment 2: proprioception + vision). (**e**) Hand positions during learning and recall.

Two behavioral experiments were conducted on altogether N = 65 younger adults to characterize the spatial code of tactile pattern memory storage and retrieval. In both experiments, participants were asked to memorize 4 tactile patterns presented to their index fingertip in a 4 × 4 matrix (i.e., 16 pins) using a vibrotactile stimulator. During learning, the hands were either crossed or uncrossed (parallel). When asked to recognize the memorized patterns (4 learned, 4 new), patterns were presented when hands were either in a crossed or in an uncrossed (parallel) position. Hence, the effect of in-/congruent hand position on tactile retrieval could be compared empirically. In experiment 1, the hands were occluded from view (testing on the effect of proprioception only), whereas in experiment 2, the hands were visible and external objects were added to the experimental setup nearby the hands (testing on the effect of both proprioception and vision). In both experiments, the accuracy of tactile pattern recall was used to compare congruent and incongruent conditions (see Fig. 1 for an overview).

### Participants performed above chance level in experiment 1 (effect of proprioception)

In experiment 1, hands were occluded from view both during learning and during recall. Learning and recall either took place in congruent or incongruent hand positions. Experiment 1 therefore tested for the effect of congruent or incongruent proprioceptive information. To test if overall tactile pattern recall was successful but not approaching ceiling, participants’ mean accuracy of pattern recall across conditions (congruent, incongruent) was computed and compared against chance level (5-alternative forced choice, chance level: 20%) using a one-sample t-test. The result shows that the overall mean accuracy is significantly higher than chance level with a large effect size, but does not approach ceiling (mean accuracy = 45.60%, t(28) = 10.832, p = 1.60*10^–11^, wilcox_p = 3.93*10^–6^, Cohen’s d = 2.01).

### No evidence for an effect of hand position on tactile pattern recall

**Fig. 2:**
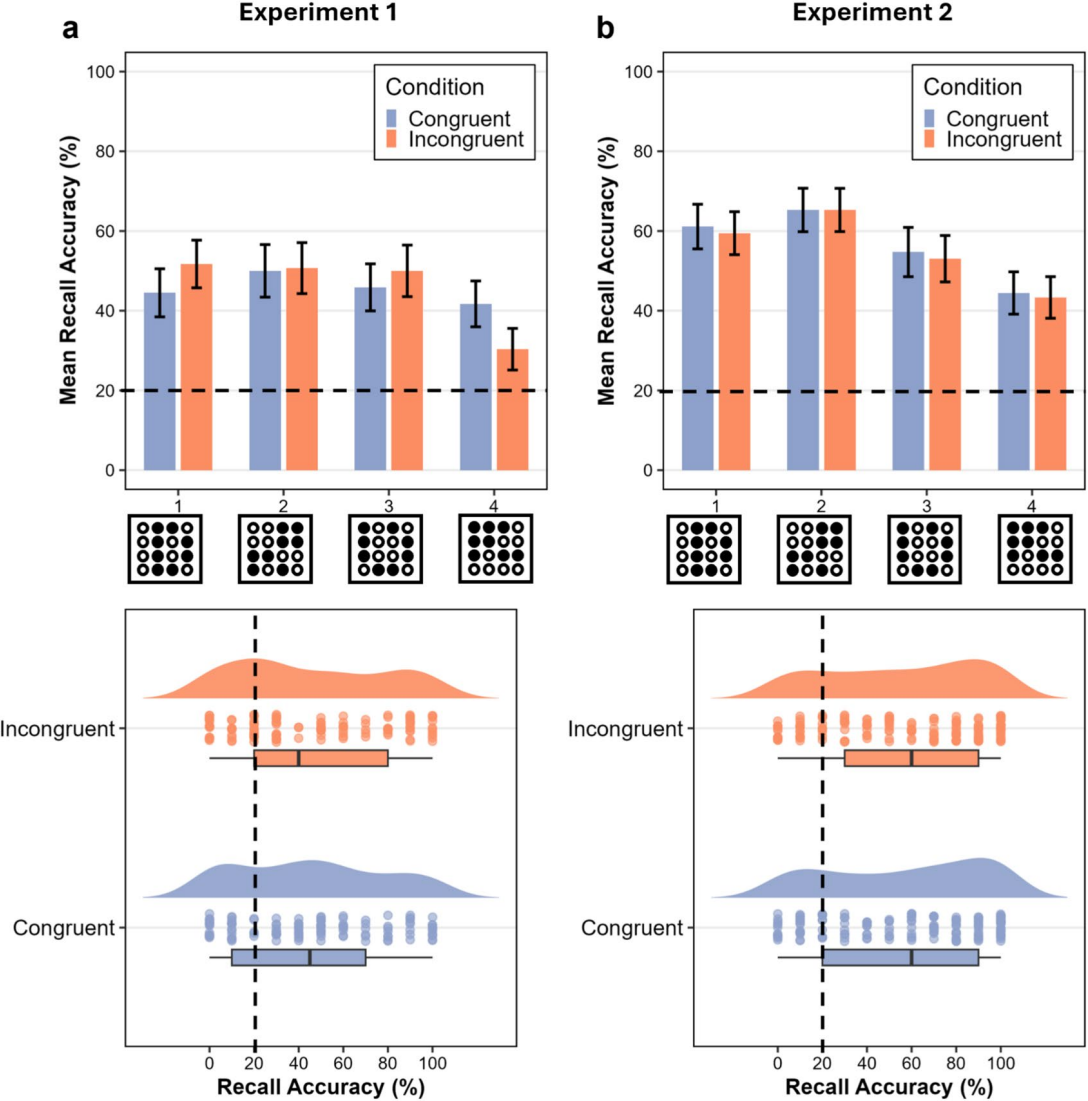
No evidence for an effect of hand position and visual context on tactile pattern recall. (**a**) Results of experiment 1 (hands occluded from view, effect of proprioception). *Top*: Plot shows mean and SE of accuracy of pattern recall for each pattern (1,2,3,4) and condition (congruent, incongruent hand position). *Bottom*: Raincloud plot shows accuracy of pattern recall for congruent and incongruent conditions along with box plots and distribution plots. (**b**) Results of experiment 2 (hands visible, effect of proprioception + vision). *Top*: Plot shows mean and SE of accuracy of pattern recall for each pattern (1,2,3,4) and condition (congruent, incongruent hand position and visual context). *Bottom*: Raincloud plot shows accuracy of pattern recall for congruent and incongruent conditions along with box plots and distribution plots. The black dotted line in each plot represents the chance level at 20%.

To test for the effect of (proprioceptive) congruency on retrieval accuracy, an ANOVA was computed with congruency (congruent, incongruent) and pattern (1,2,3,4) as within-subject factors, and % accuracy as the dependent variable. A main effect of congruency would suggest that the hand position has a significant influence on learning accuracy. The factor of pattern was integrated to test if this would be the case for specific tactile patterns only (i.e., interaction between congruency and pattern). Reversely, a missing main effect of congruency would indicate that tactile encoding is not significantly influenced by hand position. Results show that there is no main effect of congruency (F(1,203) = 0.321, p = 0.572, η^2^p = 0.00158) or pattern (F(3, 203) = 1.890, p = 0.133, η^2^p = 0.02720), and there is also no interaction between congruency and pattern (F(3, 203) = 1.150, p = 0.331, η^2^p = 0.01670) on the accuracy of pattern recall (see Fig. 2a).

Given the ANOVA does not reveal a significant main effect of congruence, next, we tested the assumption for a difference between the congruent and incongruent conditions using Bayesian Regression analyses. The model fit for the accuracy of pattern recall among participants accounted for random effects. The intercept estimate is 6.11, meaning the accuracy deviated by about 6.11% around the group mean with a 95% Credible Interval (CrI) between 0.42 and 13.04. A Rhat value of 1.00 suggests that the chains converged well, and the high effective sample size (Bulk_ESS = 1138, Tail_ESS = 1628) indicates that the model is stable^28^. Fixed effects were analysed by fitting the model to the accuracy for the two congruency conditions, four patterns and their interaction as regression coefficients using the mean accuracy of the congruent condition, and pattern 1 as a baseline (intercept). The baseline posterior mean estimate (β_0_) is 46.41 with a 95% credible interval of 39.49 to 53.19, indicating that the baseline accuracy is 46% with plausible values between 40 and 53%. Compared to baseline, the incongruent condition shows an estimate (β) of 0.48, and a CrI of -6.12 to 7.21 (see Table 1). Given the CrI encompasses zero, this does not provide evidence towards the existence of an effect.

**Table 1:** Results of Bayesian regression for experiment 1. The model was fit using a student-t distribution. Congruency with 2 levels and patterns with 4 levels were the within-subject factors, subject was the random factor. A total of 232 observations from 29 participants of experiment 1 were used. Variation due to random effects was assessed by fitting the model to mean accuracy of pattern recall for all participants. The effects of congruency and patterns was assessed by fitting the model to the mean accuracies of pattern recall for each condition. The baseline mean accuracy is chosen for congruent and Pattern1. Est.Error: Estimate Error, l-95% CI: Lower 95% Credible Interval, u-95% CI Upper 95% Credible Interval, ESS: Effective Sample Size.

Term	Estimate	Est.Error	l-95% CI	u-95% CI	Rhat	Bulk_ESS	Tail_ESS
Random effects, Group: Subject							
sd(Intercept)	6.11	3.38	0.42	13.04	1.00	1138	1628
Fixed effects (regression coefficients)							
Baseline	46.41	3.48	39.49	53.19	1.00	4302	2810
Incongruent	0.48	3.42	-6.12	7.21	1.00	4865	2743
Pattern2	2.12	3.79	-5.38	9.36	1.00	5159	2728
Pattern3	0.49	3.67	-6.57	7.62	1.00	5672	2944
Pattern4	-4.70	3.76	-12.04	2.52	1.00	5997	3286
Incongruent × Pattern2	0.82	4.19	-7.41	9.19	1.00	4924	2893
Incongruent × Pattern3	1.18	4.29	-7.12	9.49	1.00	5431	3277
Incongruent × Pattern4	-4.87	4.22	-12.96	3.37	1.00	5055	2912

Taken together, even though participants could reliably remember the tactile patterns when hands were occluded from view, ANOVA and Bayesian analyses do not support the hypothesis of an effect of an in/congruent hand position on the accuracy of tactile pattern recall when the hand was occluded from view during retrieval.

### Participants performed above chance level in experiment 2 (effect of proprioception + vision)

In experiment 2, hands were not occluded from view but were visible both during learning and during recall, and placed into a rich visual context (i.e., sand and seashell, rock and stones). Learning and recall either took place in congruent or incongruent hand positions. Experiment 2 therefore tested for the effect of congruent or incongruent proprioceptive plus visual information. The same analyses were performed as in experiment 1. Mean accuracy of pattern recall was compared to chance level (5-alternative forced choice, chance level: 20%) using a one-sample t-test. The result shows that the overall mean accuracy is significantly higher than chance level with a large effect size, yet not approaching ceiling (mean accuracy = 55.83%, t(35) = 10.688, p = 1.44*10^–12^, wilcox_p = 2.80*10^–7^, Cohen’s d = 1.78).

### No evidence for an effect of hand position or visual context on tactile pattern recall

In experiment 2, congruency was defined with respect to both hand position and visual context. Congruent conditions implied the same hands position and the same visual context during learning and recall, whereas incongruent conditions implied different hand positions and different visual context during learning and recall. The ANOVA with the factors of congruency (congruent, incongruent) and pattern (1,2,3,4) revealed no significant effect of congruency (F(1,245) = 0.0029, p = 0.957, η^2^p = 1.18*10^–5^), and no significant interaction between congruency and pattern (F(3,245) = 0.0298, p = 0.993, η^2^p = 3.65*10^–4^) on accuracy. There is, however, a significant main effect of pattern (F(3,245) = 7.670, p = 6.43*10^–5^, η^2^p = 8.58*10^–2^). Post hoc analysis revealed that the variance difference is driven by Pattern 4, the accuracy for which is significantly lower than the accuracy for both Pattern 1 and Pattern 2 across conditions, with a large effect size, Cohen’s d^29^, of 1.23 and 1.61, respectively (see Fig. 2b).

We conducted Bayesian Regression analyses to test for the likelihood of a difference between congruent and incongruent recall. The intercept estimate is 18.45, meaning the accuracy deviated by about 18.45% around the group mean with a CrI between 13.42 and 25.09. A Rhat value of 1.00 suggests that the chains converged well, and the high effective sample size (Bulk_ESS = 1287, Tail_ESS = 1709) indicates that the model is stable. For fixed effects, similar to that in experiment 1, the mean accuracy of the condition, congruent and Pattern1 was considered the baseline mean accuracy (intercept). The β_0_ is 57.20 with a CrI of 49.60 to 65.14, indicating that the baseline accuracy is 57.20% with plausible values between 49.60% and 65.14%. Compared to baseline, the incongruent condition shows a β of -0.35 and a CrI between -6.47 and 5.60 (see Table 2). Given the CrI encompasses zero, this does not provide evidence towards the existence of an effect.

**Table 2:** Results of Bayesian regression for experiment 2. The model was fit using a student-t distribution. Congruency with 2 levels and patterns with 4 levels were the within-subject factors, subject was the random factor. A total of 288 observations from 36 participants of experiment 2 were used. Variation due to random effects was assessed by fitting the model to mean accuracy of pattern recall for all participants. The effects of congruency and patterns was assessed by fitting the model to the mean accuracies of pattern recall for each condition. The baseline mean accuracy is chosen for congruent and Pattern1. Est.Error: Estimate Error, l-95% CI: Lower 95% Credible Interval, u-95% CI: Upper 95% Credible Interval, ESS: Effective Sample Size.

Term	Estimate	Est.Error	l-95% CI	u-95% CI	Rhat	Bulk_ESS	Tail_ESS
Random effects, Group: Subject							
sd(Intercept)	18.45	3.01	13.42	25.09	1.00	1287	1709
Fixed effects (regression coefficients)							
Baseline	57.20	3.95	49.60	65.14	1.00	2407	3090
Incongruent	-0.35	3.11	-6.47	5.60	1.00	4835	3097
Pattern2	4.90	3.42	-1.83	11.47	1.00	5088	2875
Pattern3	-2.35	3.52	-9.25	4.44	1.00	5243	3011
Pattern4	-8.58	3.64	-15.76	-1.38	1.00	4960	3041
Incongruent × Pattern2	1.63	4.10	-6.48	9.40	1.00	4958	3199
Incongruent × Pattern3	-1.13	3.98	-8.89	6.67	1.00	4962	3112
Incongruent × Pattern4	-2.84	4.08	-10.61	5.07	1.00	4608	2863

Taken together, participants could reliably remember the tactile patterns when hands were visible and surrounded by a rich visual context, but ANOVA and Bayesian analyses do not support the hypothesis of an effect of an in/congruent hand position and in/congruent visual context on the accuracy of tactile pattern recall.

## Discussion

We here investigate the spatial code of tactile memories by testing whether tactile pattern memories are encoded in a body-centered or in an external reference frame. For this, we performed two experiments where right-handed participants memorized tactile patterns presented to their right index fingertip using the crossed-hands paradigm. The accuracy of pattern recall was compared between congruent and incongruent recall conditions when the hand was occluded from view (experiment 1: testing for proprioception), and when the hand plus a visual context surrounding the hand was visible (experiment 2: testing for proprioception + vision). The learned tactile patterns were neither visible in Experiment 1 nor in Experiment 2. We hypothesised that, if tactile pattern memories are spatially encoded in an external reference frame, by integrating tactile, proprioceptive, and visual information, the accuracy of pattern recall would be significantly higher when the hands are in congruent compared to incongruent conditions. Conversely, if no effect of congruency can be proven, external encoding would not be supported, and this data would be in accordance with encoding in a body-centered reference frame. Our data supports the latter view and does not provide evidence for an influence of hand position (crossed, parallel) and visual context on tactile pattern recall in a scenario where tactile patterns are presented passively to the fingertip. This is consistent with a body-centered encoding of passively learned tactile pattern memories according to which neither the proprioceptive nor the visual information is informative for solving the task.

Prior experiments have shown that there is a conflict between body-centered and external reference frames when the hands are crossed^30,31^. It has been revealed in a series of temporal order judgment tasks that tactile stimulation, encoded in a body-centered reference frame initially, is remapped into an external reference frame^7,11,32^. However, the influence of this conflict paradigm on tactile memory has so far not been investigated. In experiment 1, we hypothesized that altering hand position during retrieval would disrupt the retrieval of tactile pattern memories if encoded in an external reference frame, but would not influence the retrieval of tactile pattern memories if encoded in a body-centered reference frame. In this experiment, accuracy did not approach ceiling levels, so the task was generally perceived as difficult. Nevertheless, no significant difference was observed between the recall accuracies of congruent versus incongruent conditions.

Past research has evidenced the importance of vision in the remapping of tactile stimulation^33^, also in cases where the crossing of hands does not affect the performance of congenitally blind participants as compared to those with early vision. In experiment 2, we allowed vision of the hand in addition to vision of a scene and external objects near the hand during learning and retrieval. As in experiment 1, proprioception was modulated by having the hand in either a crossed or uncrossed position. We expected that vision of the hand would generally facilitate learning via spatial attention to the hand and sharpening of tactile receptive fields^34–36^. Indeed, spatial remapping effects are task-dependent, i.e., task context influences spatial processing of touch^5^. One study used a short-term memory task and inferred that increasing memory load reduces the hand-crossing effect, thereby reducing spatial remapping^37^. However, even though learning was generally facilitated by seeing the hand, there was again no significant difference in the recall accuracy between congruent and incongruent conditions in experiment 2. In both experiments, Bayesian regression analyses did not provide evidence for the existence of an effect of congruency. The hypothesis of an external reference frame in which tactile pattern information is encoded and retrieved was therefore not confirmed by our study. This is consistent with the hypothesis that encoding and retrieval takes place in a body-centered reference frame. Future studies with larger sample sizes and a modified experimental design that directly tests for body-centered encoding (e.g., by testing on somatotopic specificity or tactile interference) will have to be conducted to support this theory.

With respect to potential neuronal networks underlying this effect, the PPC has been shown to be involved in the remapping of tactile events^13,27,38^, and in the processing of tactile memories^15,39^. Given we do not see evidence in our data that remapped tactile memories are recalled, our data favors the view that somatosensory networks, such as SI, SII and/or the insula cortex, are involved in tactile memory encoding^22,23^. In agreement with this view, an fMRI study on working memory of tactile stimuli has reported that SI retains the tactile information whereas PPC retains the spatial information^26^. In a scenario where only the tactile information is learned, such as when encoding fine-grained pattern at the fingertip, it can be speculated that the information pathway from SI to SII and the insula cortex, in interaction with the medial temporal lobe, mediates pattern recall. However, future studies will have to test this using neuroimaging methodology.

Tactile memories do not only guide object recognition but also contribute to body memories and potentially to the development of somatic symptoms, memory intrusions, or flashbacks^3^. It is interesting that somatic symptoms are often topographic and occur at the same body location^3,40^. It is therefore possible that tactile memories encoded in a body-centered reference frame contribute to topographically organized somatic symptoms. However, given body memories are shaped by body movement, interoceptive states, and pain, more research would be needed to embed our findings into more complex bodily processing that characterizes everyday life.

When we assume tactile pattern memories to be encoded in a body-centered format, mediated by areas that have a topographic mapping profile (i.e., SI, SII, insula cortex), the next step would be to investigate if tactile memories are indeed stored in a topographic format, i.e., if memories of tactile stimulations at neighbouring points on the skin are mapped to nearby cortical regions compared to tactile stimulation at distant points on skin (e.g., distant versus neighbouring fingers). A topographic organization has been shown in tactile working memory with behavioral experiments on vibration discrimination^41^, and for tactile learning with behavioral experiments on vibration discrimination, pressure and surface roughness^42^. It has also been shown that observed tactile experiences are encoded topographically in SI when the task is to rate tactile features of the observed touch^43^. Similar experiments can be performed on learning and retrieval of tactile patterns to investigate the a/topographic organization of tactile memories, providing helpful insights into the neuronal mechanisms underlying the storage and retrieval of tactile memories.

It is worth highlighting drawbacks of the present study. First, we used a behavioral setup where the involvement of specific neuronal networks can only be speculated on. However, given the time and effort it takes to transfer the behavioral crossed-hand paradigm to an fMRI experiment (with crossing hands being hampered by the lying position in the MRI), a behavioral setup provides first indications of which experimental paradigm may activate critical networks. Second, our conclusions are based on no detection of a difference between congruent and incongruent conditions, where no difference does not support the existence of an effect. Future experiments could investigate the specific involvement of lesions in SI, SII and/or the insula cortex on performance in this paradigm to provide causal evidence for the activation of a body-centered reference frame mediated by these networks, could investigate the topographic architecture of this effect, and/or test for tactile interference effects. We regard this study as a valuable first step guiding future research on detailing the mechanisms that underlie the spatial encoding of touch, including the involved neuronal networks.

Third, there is a significant effect of pattern in experiment 2, driven by reduced performance to recall Pattern 4. This raises questions about stimulus equivalence as pattern variability may interact with the congruency effects, having potential implications on accuracy of pattern recall. Statistically, however, the interaction between condition and pattern is not significant. In addition, there is no significant difference between patterns in experiment 1, where the same patterns were used. Why Pattern 4 was more difficult particularly in experiment 2 is at that point not clear to us, but may be addressed by future research.

Fourth, in experiment 2, the effect of congruency simultaneously manipulated hand position and visual context. Whereas experiment 1 manipulated proprioception only, experiment 2 manipulated both proprioception and vision. This modular approach allowed us to first test for the isolated effect of proprioception, and to subsequently test for the combined effect of proprioception and vision. However, this experimental design also implies that experiment 2 does not allow a dissociation between the effects of proprioception and vision, i.e., the effect of visual congruency was not tested in isolation. Theoretically, it is possible that only manipulating vision (without proprioception) influences tactile encoding and retrieval. Whereas based on the obtained results, we think that this is unlikely, it nevertheless remains unaddressed by the present paradigm and needs to be addressed by future research.

Finally, it should be noted that whereas in an everyday situation, hand position but in particular visual context can be relevant to the tactile encoding (i.e., position of hand in bag when searching for key, contour of door when trying to open door in the dark), this is not the case here. In our experiments, proprioceptive and visual context were irrelevant to the task. Therefore, here, we tested whether encoding in a body-centered reference frame would occur when other information is irrelevant or even distracting, but does not investigate multisensory memory formation with more than one modality being relevant to the task.

In conclusion, the study shows that there is no significant difference in retrieving tactile patterns in congruent or incongruent hands positions, and with congruent or incongruent visual context in reference to the learning condition. This is consistent with the interpretation that passively learned tactile pattern memories are encoded in a body-centered reference frame, and that the information that is retrieved after learning is likely remapped into external space but not used for storage and retrieval. Although the PPC is known to be involved in processing of tactile information, one may hypothesize based on this data that SI, SII and insular cortex mediate the processing of tactile memories, which warrants to be investigated precisely in future research.

## Methods

### Participants

Altogether N = 65 younger healthy adults, aged between 18 and 30 years, were recruited for both experiments. All participants had normal or corrected vision, and no limb-related disorders. None of the participants had any diagnosed neurological or psychiatric conditions and none of them were on any medications that would affect their cognitive abilities. Handedness was assessed using the Edinburgh Handedness Inventory^44^. In experiment 1, n = 36 participants (mean age ± SD = 23.08 ± 2.68 years, age range: 18 to 28, 9 males) took part. n = 6 participants had to be excluded due to technical difficulties of data recording, n = 1 participant was excluded due to handedness (ambidextrous). Data from n = 29 participants (mean ± SD = 22.87 ± 2.71 years, age range: 18—28 years, 6 males, handedness score ± SD = 86.78 ± 13.40) were included in the analyses of experiment 1. In experiment 2, n = 47 participants (mean ± SD = 24.10 ± 2.72 years, age range: 20—29 years, 12 males) took part. n = 2 participants were excluded due to handedness (ambidextrous), n = 2 participants were excluded because of technical difficulties of data recording, and n = 7 participants were excluded because they could not fulfill the learning criteria (see below). Data from n = 36 participants (mean ± SD = 24.35 ± 2.65 years, age range: 20—29 years, 8 males, handedness score ± SD = 81.08 ± 15.91) were included in the analyses. All participants included in the final analyses were right-handed.

Informed consent was obtained from all participants. The study was approved by the Ethics Committee of the Medical Faculty of the Eberhard-Karls University of Tübingen, Germany (Ethics No.: 651/2022BO1). The study was conducted in accordance with the approved ethical guidelines. Participants were given 8€ per hour as compensation for their participation.

### Stimulus material: tactile patterns

Tactile patterns were presented to the fingertip of the right index finger of each participant using two combined modules from the QuearoSys Piezostimulator (Piezostimulator, Quaerosys, St. Johann, Germany) controlled via a Thinkpad notebook and Matlab (R2015b) via the Psychtoolbox (last update: 07/02/2013)^45^. The stimulation modules consisted of 16 pins (each module consisted of 8) arranged in a 4 × 4 matrix with 2.5 mm spacing between each pin (see Fig. 1a). Each pin on the modules could be controlled separately and is able to reach 4096 different heights between 0 mm and 1.5 mm with a 0.5 ms timing accuracy^45^. 8 pins were used to generate one pattern. There were in total 12 patterns designed and presented in both experiments (see Fig. 1a). In each experiment, participants learned 4 patterns, and had to discriminate them from 4 new patterns. The pins vibrated at a sinusoidal frequency of 16 Hz to present the tactile patterns^46^. The stimulus duration for each pattern was 1000 ms. The interstimulus interval was 2000 ms.

### Experimental setup

The hands of the participants were always placed in front of them, palms down, on a table. In some experimental conditions, they were placed parallel (P) to each other, in other experimental conditions, they were placed in a crossed position (C) (see Fig. 1). Congruency in experiment 1 is defined as recall of patterns with hands in the same positions as in the learning phase, and incongruency is defined as recall of patterns with hands in a different position compared to the learning phase (effect of proprioception). In experiment 2, the hands and the context surrounding the hands were visible. Images were shown on a screen that was placed below participants’ hands (showing rocks and a beach, respectively), and external objects were added to reveal a 3D effect (stone and seashell, respectively). Congruency in experiment 2 is therefore defined as recall of the patterns with the hands in the same position as in the learning phase in addition to being presented with the same visual context as in the learning phase, whereas incongruency is defined as recall of patterns with hands in a different position compared to the learning phase in addition to being presented with a different visual context as in the learning phase (effect of proprioception + vision).

### Experimental setup: experiment 1 (effect of proprioception)

The setup for experiment 1 is shown in Fig. 1b. In experiment 1, a stimulation module was placed on the table under the right index fingertip to provide tactile stimulation. The hands were covered using an opaque black cardboard box to occlude them from view. The foot switch, consisting of three buttons, was placed below participants’ feet to record their foot responses. A display monitor was positioned in front of the participant to provide them instructions on which switch corresponds to which response and the feedback on their responses during the learning phase.

### Experimental setup: experiment 2 (effect of proprioception + vision)

The setup for experiment 2 is shown in Fig. 1c. It is similar to the setup of Experiment 1 with the following modifications: (1) The hands were not occluded from view (i.e., no box covering the hands). (2) A display monitor was positioned flat on the table, screen up, below participants’ hands. The screen was covered using a transparent acrylic sheet to protect the screen from the touch. A velcro strip was glued on the sheet on both hand sides to ensure constant hand position. During stimulus presentation, learning, and recall, the right side of the screen showed a scenery with sea and sand, whereas the left side showed a scenery with rocks and mountains (see Fig. 1c). (3) A stone (right) and a seashell (left) were glued on the acrylic sheet to add a textural 3D context during learning phase and recall phase. (4) The stimulator module was taped to the participants’ right index finger.

### Experimental task

Both experiments 1 and 2 consisted of three phases: the stimulus presentation phase, the learning phase, and the recall phase (see Fig. 1d).

### Experimental task: experiment 1

**Fig. 3:**
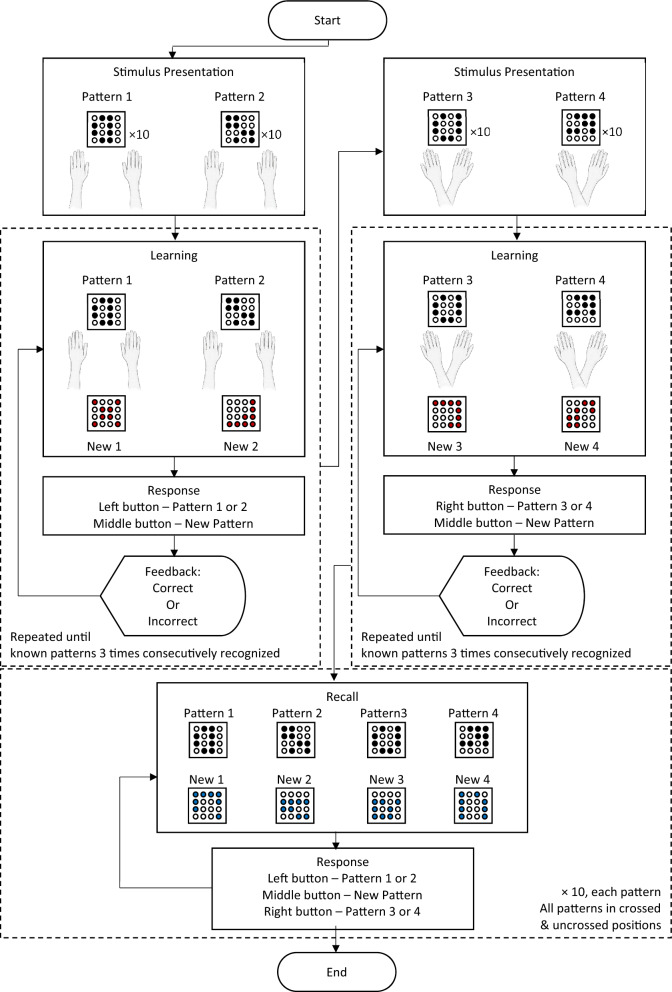
A flowchart describing the experimental task in an example trial for experiment 1. The experimental task was performed in three phases, the stimulus presentation phase, the learning phase and the recall phase. The experiment started with the stimulus presentation phase of the first two patterns (black dots). Each pattern was presented 10 times individually, one after the other. This was followed by the learning phase for these two patterns. The hand position was the same for both the stimulus presentation phase and the learning phase (parallel, P, or crossed, C). During the learning phase, the participants were asked to discriminate between two known patterns (black dots) and two new patterns (red dots), presented in a pseudorandom order. For each pattern, participants were asked to respond using the foot switch by pressing the left button once or twice for known patterns and the middle button for new patterns. After the response, they were given feedback showing if their response was correct or incorrect. The learning phase was repeated until participants correctly identified the known patterns 3 times consecutively. Each pattern was repeated a maximum of 10 times. After the learning phase for the first two patterns, stimulus presentation and learning phases were repeated for the remaining two patterns, however, with hands in the respective other position (if first P, then now C, and vice versa). After stimulus presentation and learning of all four patterns (two in P and two in C), the experiment continued with the recall phase, where participants were asked to identify the four learned patterns (black dots) and four new patterns (blue dots) in congruent and incongruent hand position (congruent: learned in P and recalled in P, learned in C and recalled in C, incongruent: learned in P and recalled in C, or learned in C and recalled in P). No feedback was provided. Each pattern was presented 10 times in each hand position. The data recorded during the recall phase was used for the analyses.

An example trial to describe the experimental task for experiment 1 is shown in Fig. 3. The experiment began with the stimulus presentation phase, followed by the learning phase. During both phases, 50% of participants learned the first two patterns with hands in parallel (P), and 50% participants with hands crossed (C). During the stimulus presentation phase, each pattern was presented 10 times, with the name of the pattern being displayed on the screen (e.g., “Pattern 1”, “Pattern 2”). This was followed by the learning phase for those two patterns. During the learning phase, participants were asked to discriminate between the two patterns they had learned in the stimulus presentation phase and two new patterns. The patterns were presented in a pseudorandom order. Feedback was provided in the form that participants saw on the screen if they responded correctly, or not. Participants had to correctly identify each of the memorized patterns at least 3 times in a row. If this could not be achieved when each pattern was presented a maximum of 10 times, the experiment was repeated, starting from the stimulus presentation phase. The stimulus presentation and learning phases were repeated a maximum of 3 times. If a participant did not achieve this criterion by then, the experiment was stopped and the respective data was excluded. This procedure was repeated for the other two patterns that were either presented and learned in crossed or uncrossed positions, respectively (“Pattern 3”, “Pattern 4”). For half the participants, the experiment began with the presentation of the Patterns 1 & 2, and for the other half, the experiment began with the presentation of the Patterns 3 & 4. Hand position was not changed during the stimulus presentation phase and learning phase.

In the recall phase, i.e., the actual experiment, participants were then tested on their ability to correctly identify all four patterns, and to distinguish them from four new patterns (different from the four new patterns shown in the learning phase, see Fig. 1a,d). Tactile patterns were presented in a pseudo random order. In the recall phase, hand positions changed in a way that each pattern had to be recalled 10 times in a position congruent to the learning phase, and 10 times in a position incongruent to the learning phase (see Fig. 1e). Half of the participants began the recall phase with hands in parallel position, and the other half began the recall phase with hands in crossed position. Similar to the learning phase, participants were asked to identify the presented pattern or to decide that the pattern was new, and to answer using the foot switch. There was no feedback shown to the participants on whether their answers were correct or not. The foot switch had 3 buttons. To indicate Pattern 1, the left button was pressed once, and for Pattern 2, the left button was pressed twice. To indicate Pattern 3, the right button was pressed once, and for Pattern 4, the right button was pressed twice. To indicate a new pattern, the middle button was pressed once. If the pattern presented during the recall phase was identified correctly using the foot switch, it was recorded as binary ‘1’, and if the pattern presented was identified incorrectly, it was recorded as binary ‘0’. An answer was only coded as correct if the correct pattern among the four was identified, or if a new pattern was correctly identified, respectively.

### Experimental task: experiment 2

The experimental procedure in experiment 2 was similar to the one in experiment 1. One difference was that pattern presentation was divided into 3 groups. This was done to counterbalance any effects due to presentation of the same patterns grouped together every time. During stimulus presentation, 1/3 of the participants learned patterns 1 & 2 (50% P and 50% C) and 3 & 4 (50% P and 50% C) together; 1/3 of the participants learned patterns 1 & 3 (50% P and 50% C) and patterns 2 & 4 (50% P and 50% C) together; finally, 1/3 of the participants learned patterns 1 & 4 (50% P and 50% C) and patterns 2 & 3 (50% P and 50% C) together. Similar to experiment 1, for each participant, the position of the hands remained the same during the stimulus presentation phase and the learning phase. In the recall phase, hand positions changed in a way that each pattern was recalled 10 times in a position congruent to the learning phase, and 10 times in a position incongruent to the learning phase. The foot switch was used as in experiment 1 to indicate which pattern was perceived. An answer was only coded as correct if the correct pattern among the four was identified, or if a new pattern was correctly identified, respectively. In addition, the difference to experiment 1 was that the hands were no longer occluded, and visual context was added (see explanation above).

### Statistical analyses

Pattern recall accuracy was calculated as the averaged percentage of correct responses for each participant. The accuracy was used as the dependent variable for the analyses. The chance level was 20% as the participants were instructed to choose a response from 5 potential answers (“Pattern 1”, “Pattern 2”, “Pattern 3”, “Pattern 4”, “New”).

Jupyter notebook (Python 3) and RStudio 2025.05.1 were used for statistical analyses. RStudio was also used for data visualization. Data was tested for the assumption of normal distribution with the Shapiro Wilk’s test of normality (*p* > 0.05) and the assumption of homoscedasticity using the Levene’s test of homogeneity of variance (*p* > 0.05). When the data showed deviation from normality, robustness check was performed using Wilcoxon signed-rank test.

The following same statistical analyses were performed on the data of both experiments. A one sample t-test was performed to test overall performance against the chance level of 20%. Cohen’s d with 95% confidence intervals was computed to determine the effect size with d ≤ 0.2 being small effect size, 0.2 > d ≤ 0.7 being medium effect size and d ≥ 0.8 being large effect size^29^.

To investigate the effect of congruency on tactile pattern memory retrieval, a 2 × 4 Aligned Rank Transform (ART) ANOVA with 95% significance level was computed on the accuracy of pattern recall with the factors patterns (Pattern 1, Pattern 2, Pattern 3, Pattern 4) and congruency (congruent, incongruent) as the within-subject categorical factors, and subject as random factor. The factor of pattern was integrated given recent research indicates a non-linear representation of touch in S1^47^. ART is a nonparametric factorial data analysis method in which there is a preprocessing step of aligning the data before applying averaged ranks. ANOVA procedures can be performed on the aligned data using f-statistic, p-value and effect size^48^. In case of significant effects of the factors on accuracy of pattern recall, a post hoc analysis was performed on the factor of interest using Bonferroni adjusted pairwise comparison with effect size, Cohen’s d. Congruency was defined as retrieval of the patterns with hands in the same positions as in learning and incongruency was defined as retrieval of patterns with hands in different positions compared to learning.

To evaluate the strength of evidence for differences between congruent and incongruent conditions, we complemented frequentist null-hypothesis significance testing using Bayesian Regression and Multilevel modeling as implemented in the Stan (brms) package. The brms package is an R package that uses the probabilistic programming language Stan to implement Bayesian multilevel models^28^. This dual approach was employed to provide a more nuanced interpretation of the effects: while frequentist models provided binary significance thresholds, the Bayesian framework allowed us to quantify the posterior probability of our parameters. Furthermore, the Bayesian approach provides a more robust estimation of participant-level variance in our within-subjects design, offering a clearer quantification of the evidence for the observed effects. The effect on accuracy was predicted using a Student-t model. Although accuracy is a bounded proportion, the Student-t distribution was chosen as it offered robustness against the deviation from normality and outliers. This was further validated by a leave-one-out (LOO) cross validation, where all Pareto k estimates were good (k < 0.7) for both experiment 1 and experiment 2, indicating good fit of the model.

The pattern and congruency were specified as the within-subject factors and subject as the random factor, using the formula:

$$Accuracy \sim Congruency * Pattern + (1|Subject)$$

Everything before the ~ sign refers to the response variable. Everything after the ~ sign refers to the predictors. The + separates different effects from each other^28^. Prior distributions are specified for the model parameters, one for the intercept (baseline mean), one for the fixed-effects slope, one for the residual standard deviation and one for the degrees of freedom. The brms uses the no-U-turn sampler (NUTS)^49^ to fit the models. The brms fits the model using 4 chains, 4 cores, each with 200 iterations, out of which the first 1000 were warmup samples used to calibrate the sampler, resulting in a total of 4000 posterior samples. The brms summarised the data using posterior mean (estimate) and the standard deviation (estimates error) of the posterior distribution along with two-sided 95% credible intervals (CrI). The Bulk_ESS and Tail_ESS values are an estimation of the effective sample sizes from the posterior distribution that would be expected to give the standard error of the posterior mean as is obtained from the dependent samples. The Rhat value gives the information about the convergence of the algorithm. If Rhat is substantially greater than 1 (for example: > 1.1), the chains were not converged yet and needed more iterations to be run and/or stronger priors to be set^28^. In case the 95% CrI of a factor does not overlap with 0 when compared to the baseline accuracy, it implies a significant effect of that factor on the accuracy of pattern recall. In case, on the other hand, the 95% CrI of a factor does overlap with 0 when compared to the baseline accuracy, it does not indicate a significant effect to the baseline accuracy. As baseline accuracy, the congruent condition was chosen.

## Data Availability

The data analysed in the current study will be made available upon formal request to Shreyas Indurkar.
